# Supplementary material for: Predicting visual function by interpreting a neuronal wiring diagram
Source: Nature. 2024 Oct 2;634(8032):113–23. doi: 10.1038/s41586-024-07953-5 (PMC11446822; doi:10.1038/s41586-024-07953-5)
Supplement: Supplementary file 6 — CRF and ERF predictions for individual TmY4 and TmY9 cells. Analogous to Supplementary Data 3, but for TmY target types. Shown are the top four monosynaptic pathways, the strongest pathway passing through each of the top ten intermediary types (ranking from Extended Data Fig. 7), and the trisynaptic pathway Tm1–TmY–Dm3–TmY (see the section entitled Prediction of spatial normalization). [file 41586_2024_7953_MOESM6_ESM.zip › DataS4/TmY9q/720575940615973738.pdf]

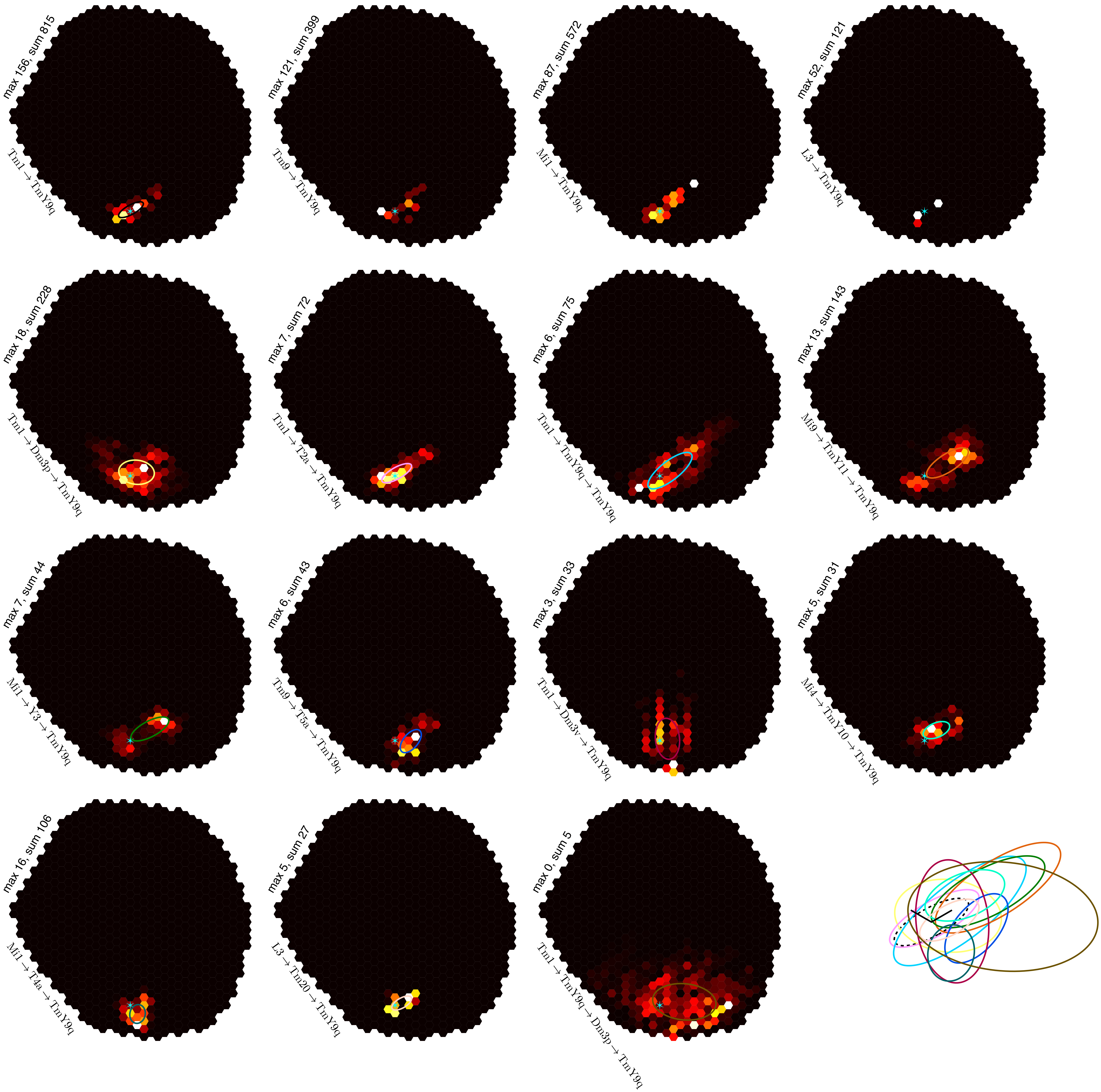
max 156, sum 815
Tm1 → TmY9q
max 121, sum 399
Tm9 → TmY9q
max 87, sum 572
Mi1 → TmY9q
max 52, sum 121
L3 → TmY9q
max 18, sum 228
Tm1 → Dm3p → TmY9q
max 7, sum 72
Tm1 → T2a → TmY9q
max 6, sum 75
Tm1 → TmY9q → TmY9q
max 13, sum 143
Mi9 → TmY11 → TmY9q
max 7, sum 44
Mi1 → Y3 → TmY9q
max 6, sum 43
Tm9 → T5a → TmY9q
max 3, sum 33
Tm1 → Dm3v → TmY9q
max 5, sum 31
Mi4 → TmY10 → TmY9q
max 16, sum 106
Mi1 → T4a → TmY9q
max 5, sum 27
L3 → Tm20 → TmY9q
max 0, sum 5
Tm1 → TmY9q → Dm3p → TmY9q
